# Establishment and Characterization of Continuous Satellite Muscle Cells from Olive Flounder (*Paralichthys olivaceus*): Isolation, Culture Conditions, and Myogenic Protein Expression

**DOI:** 10.3390/cells12182325

**Published:** 2023-09-21

**Authors:** Sathish Krishnan, Selvakumari Ulagesan, Josel Cadangin, Ji-Hye Lee, Taek-Jeong Nam, Youn-Hee Choi

**Affiliations:** 1 Institute of Fisheries Sciences, Pukyong National University, Gijang-gun, Busan 46041, Republic of Korea; sathishmbt@gmail.com; 2 Division of Fisheries Life Sciences, Pukyong National University, Nam-gu, Busan 48513, Republic of Korea; ula.selva@gmail.com; 3 Department of Fisheries Biology, Pukyong National University, Nam-gu, Busan 48513, Republic of Korea; jcadangin51@gmail.com (J.C.); lizzy825@pukyong.ac.kr (J.-H.L.)

**Keywords:** olive flounder, fish muscle cell, fish satellite cell, skeletal muscle, continuous cell line

## Abstract

Olive flounder (*Paralichthys olivaceus*) muscle satellite cells (OFMCs) were obtained by enzymatic primary cell isolation and the explant method. Enzymatic isolation yielded cells that reached 80% confluence within 8 days, compared to 15 days for the explant method. Optimal OFMC growth was observed in 20% fetal bovine serum at 28 °C with 0.8 mM CaCl_2_ and the basic fibroblast growth factor (BFGF) to enhance cell growth. OFMCs have become permanent cell lines through the spontaneous immortalization crisis at the 20th passage. Olive flounder skeletal muscle myoblasts were induced into a mitogen-poor medium containing 2% horse serum for differentiation; they fused to form multinucleate myotubes. The results indicated complete differentiation of myoblasts into myotubes; we also detected the expression of the myogenic regulatory factors myoD, myogenin, and desmin. Upregulation (Myogenin, desmin) and downregulation (MyoD) of muscle regulation factors confirmed the differentiation in OFMCs.

## 1. Introduction

Olive flounder (*Paralichthys olivaceus*), a demersal marine flatfish, is an economically important aquaculture species in Asian countries, including Korea, Japan, and China [1]. The Western Pacific temperate/subtropical coastal waters are an ideal habitat for this species, which prefers water depths of 10–100 m [2]. Although able to tolerate temperatures of 5.8–28.5 °C, olive flounder prefers 20–25 °C [3]. There is increasing demand for this oval-shaped fish cultured on the southern coast of South Korea and Jeju Island [4]. Several cell lines have been developed from the organs of olive flounder, including (FG)-9307 gill cells [5], Hirame natural embryonic cells [6], spleen and gill cells [7], embryonic cells [8], flounder brain cells [9], and muscle satellite cells [10]. Establishing stable cell lines from olive flounder is important for research and commercial cell-based seafood production.

Fish cell lines are used as model systems in the studies of virology, physiology, toxicology, immunology, genetics, oncology, developmental biology, medicine, biotechnology, epidemiology, molecular carcinogenesis, functional genomics, and for the large-scale production of biologicals in pharmacology [11,12]. The surge in the use of fish cell culture in research is mainly due to the demand to reduce or replace animal experiments. Additionally, fish cell culture allows drugs to be tested, is reproducible and economically feasible, and yields results rapidly [13]. Fish cell culture could function as the best source for animal-free seafood production through cellular aquaculture [14,15]. Cellular aquaculture primarily relies on the development of satellite skeletal muscle cells. Satellite cells are mononucleated cells that lie under the basal lamina of myofibers or are embedded within them. They are responsible for the growth, regeneration, and repair of skeletal muscle tissue. When activated, satellite cells undergo cell division and differentiation to form new muscle fibers. They can also fuse with existing muscle fibers to increase their size [16,17].

Fish cell culture benefits from in vitro growth, tolerance to hypoxia and low temperatures, and high buffering capacity. The global demand for seafood is expected to increase by 70% by 2050, but the current methods of production are unsustainable [18]. Industrialized fisheries and marine capture have decreased ocean biomass by up to 80% [19]. Nevertheless, aquaculture is seen as an alternative to wild capture. However, aquaculture is not a sustainable solution to the problem of food security because it requires large amounts of land, water, and feed, and it also relies on wild-caught fish for feed for aquaculture animals [18,19]. Pollutants (heavy metals, microplastics, and chemicals) threaten aquatic food safety and pose a major challenge for national and international agencies. These pollutants can harm health if consumed in toxic quantities [20,21,22]. Because the ocean is at risk, cellular-based seafood production would be useful. However, this requires studies on fish muscle cell cultivation, differentiation, optimization of fish-cell culture conditions, serum-free media, and bioreactor designs for large-scale production.

Cellular aquaculture is a new and emerging field that aims to produce seafood from cell cultures rather than from whole fish [15]. However, cellular aquaculture is still in its early stages of development, and there are several challenges that need to be addressed before it can become a viable commercial reality [23]. This could help to address food security issues by improving production efficiency, expanding scale, and establishing precise cultivation conditions. However, there is currently a limited availability of skeletal muscle cell lines that are suitable for producing cultured fish meat or serving as a research model. This is especially true for commercially important fish species [24]. In recent years, there has been a surge in the establishment and characterization of new fish cell lines representing diverse fish species and tissue types. This has generated considerable excitement within the scientific community. In view of this, primarily skeletal muscle satellite cells from olive flounder were isolated, and the optimal growth conditions and myogenic protein expression were determined.

## 2. Materials and Methods

### 2.1. Sample Collection and Maintenance

Olive flounder (*Paralichthys olivaceus*) was obtained and used for muscle cell isolation. Juvenile olive flounders (body weight: 8.0–15.97 g; total length: 95–119 mm) sourced from a private farm in Boryeong, South Chungcheong Province, were stocked in five 450-L seawater-filled fiberglass tanks located at the Institute of Fisheries Science, Pukyong National University, Busan, Republic of Korea. Upon arrival, fish exhibiting any disease, such as body ulcerations, exophthalmia, and abnormal swimming behavior, were removed from the cohort. Normal and healthy-looking individuals were subjected to a water bath containing 100 ppm oxytetracycline (Samyang OTC-24, Pocheon, Republic of Korea) for an hour as a prophylactic treatment before being stocked in tanks. Fish were reared in tanks continuously supplied with aeration and freshly pumped natural seawater via a flow-through culture system. This seawater was filtered through progressively smaller filters (50 µm, 25 µm, 1 µm) and sterilized with UV light. Fish were hand-fed twice daily (9:00 A.M. and 5:00 P.M.) with commercial olive flounder feed (Flounder Super Plus No. 9, Suhyup Feed, Republic of Korea), ad libitum, until apparent satiation. The range of temperature, salinity, pH, and dissolved oxygen level during rearing was as follows: 15.00–25.00 °C, 28.00–35.00 PSU, 7.70–8.28, and >5.00 mg, respectively. Parameters were manually measured using a Portable multimeter AM70 (Apera Instruments, Columbus, OH, USA).

### 2.2. Medium Preparations

#### 2.2.1. Isolation Medium

Isolation medium was prepared using L-15 medium supplemented with 3% fetal bovine serum (FBS; Gibco; Lot number: 26140079), 0.8 mM CaCl_2_, 2 mM glutamine, and 100 µg/mL (1%) penicillin/streptomycin (Gibco; Billings, MT, USA Lot number: 15070063).

#### 2.2.2. Complete Growth Medium

Complete growth medium was prepared using L15 supplemented with 20% FBS Gibco; Lot number: 26140079), 0.8 mM CaCl_2_, 2 mM glutamine, and 100 µg/mL (1%) penicillin/streptomycin (Gibco; Lot number: 15070063).

#### 2.2.3. Differentiation Medium

Differentiation medium was prepared using L15 supplemented with 2% horse serum Gibco; Lot number: 16050122), 0.8 mM CaCl_2_, 2 mM glutamine, and 100 µg/mL (1%) penicillin/streptomycin (Gibco; Lot number: 15070063).

### 2.3. Enzymatic Isolation of Primary Cells

For enzymatic cell isolation, sub-adult olive flounder (body weight: 320 g; total length: 230 mm) fish were anesthetized and euthanized with 0.02% (200 mg/L) MS-222 (ethyl 3-aminobenzoate methanesulfonate; Sigma-Aldrich, St. Louis, MO, USA). Further, the euthanized fish were then washed in double-distilled water with 0.5% sodium hypochlorite (bleach) to remove contaminants and oils. The bleach was removed by washing the fish three times with phosphate-buffered saline (PBS: pH-7.4; Gibco; Lot no: 10010023). The fish were then dipped in ethanol for 30 s to prevent bacterial or fungal contamination. The ethanol was removed by rinsing the fish twice with PBS. The internal organs were removed using sterile tweezers and forceps, and the skeletal muscles were removed using a sterile scalpel and placed in a sterile Petri dish.

Soft muscle tissue (5 g) was transferred to a biological safety cabinet and washed twice with PBS containing 1% of penicillin/streptomycin (Gibco; Lot number: 15070063), antibiotic and antimycotic (Gibco; lot number: 15240062). The tissue was minced using scissors in a sterile Petri dish. The minced skeletal muscle was enzymatically digested using 10 mL of 5 mg/mL collagenase type IV (Gibco; lot number:17100017) solution and incubated in a sterile 50 mL conical tube for 45 min with rotation at 200 rpm at 4 °C. Isolation medium (20 mL) was added after incubation to terminate collagenase activity. Next, the cell suspension was filtered through 100, 70, and 40 µm nylon strainers (Thermo Fisher Scientific, Waltham, MA, USA). The filtrate was centrifuged at 1000× *g* for 10 min at 4 °C, and the supernatant was discarded. The pellet was resuspended in 5 mL of PBS, and 5 mL of Ficoll^®^ Paque Plus solution (Sigma-Aldrich; Lot number: GE17-1440-02) were added to the 15 mL centrifuge tube, which was centrifuged at 4 °C for 45 min at 1400× *g*. Primary fish cells were collected from the top layer and washed with PBS to remove Ficoll prior to resuspension in 10 mL of the complete growth medium. Cells were counted using an automated cell counter (LUNA-FL™ Dual Fluorescence Cell Counter, Logos Biosystems, Anyang, Gyeonggi-do, Republic of Korea), plated on 100 mm culture dishes with the seeding density of 2.88 × 10^6^ cells/mL, and incubated at 28 °C in a sterile tissue culture incubator without CO_2_. Half of the complete growth medium was changed every 2 days.

### 2.4. Explant Isolation of Primary Cells

Fish tissue was minced in a sterile Petri dish using a sterile dissecting blade and scissors. Approximately 25 tissue fragments (1–2 mm^3^) were separately explanted in a 100 mm cell-culture dish in 200 μL of FBS and allowed to attach for 8 h at 28 °C; L-15 complete growth medium was added. The culture dish was incubated at 28 °C, and the complete growth medium was replaced every 3 days. The culture dishes were monitored daily for attachment, spreading, and proliferation using an inverted microscope.

### 2.5. Optimal Growth Conditions

#### 2.5.1. Serum Concentration

The growth rate was assessed over 8 days using 10, 15, and 20% FBS. In order to prepare a complete medium with different concentrations of FBS, L15 medium was supplemented with different FBS concentrations (10, 15, and 20%) along with the 0.8 mM CaCl_2_, 2 mM glutamine, and 100 µg/mL (1%) penicillin/streptomycin (Gibco; Lot number: 15070063). Cells were seeded in a six-well plate at 1 × 10^5^/well at passage 6. Cells were trypsinized (detached using 0.25% trypsin-EDTA; Gibco; Lot number: 25200056) on alternate days and counted using a LUNA-FL™ Dual Fluorescence Cell Counter.

#### 2.5.2. Temperature

Olive flounder muscle satellite cells (OFMCs) were seeded at 1 × 10^5^/well at passage 6 in six-well plates in a complete growth medium. The cells were incubated at 20, 24, and 28 °C for 8 days. Cells were trypsinized and detached on alternate days and counted using a LUNA-FL™ Dual Fluorescence Cell Counter (Logos Biosystems).

#### 2.5.3. Salts

OFMCs were cultured in the presence of NaCl (2 mM) and CaCl_2_ (0.8 mM) in complete growth medium. Cells were seeded at 1 × 10^5^/well at passage 6 and incubated for 8 days in six-well plates. Cells were removed, trypsinized, and detached for enumeration in triplicate on alternate days.

#### 2.5.4. Growth Factors

Insulin-like growth factor (IGF; Sigma), epidermal growth factor (EGF; Peprotech), and basic fibroblast growth factor (bFGF; peprotech) were used at 10 ng/mL in complete growth medium. Cells were seeded at 1 × 10^5^/well at passage 6 and incubated for 8 days at 28 °C. Cells were removed in triplicate and trypsinized and detached for enumeration using the LUNA-FL™ Dual Fluorescence Cell Counter.

### 2.6. Subculture and Differentiation

OFMCs were cultured in complete growth medium (L-15 medium supplemented with 20% FBS (Gibco; Thermo Fisher Scientific, Waltham, MA, USA), 100 U/mL penicillin, and 100 μg/mL streptomycin (Gibco; Lot number: 15070063)) at 28 °C. Myoblasts were grown to 70–80% confluence in culture dishes (100 mm), trypsinized (detached using 0.25% trypsin-EDTA; Gibco; Lot number: 25200056), and seeded (4 × 10^4^ cells/well) into six-well culture plates. Cells were grown to 70–80% confluence in L-15 medium supplemented with 20% FBS for 24 h, and the medium was replaced with differentiation medium (L-15 medium containing 2% horse serum) to induce differentiation into myotubes. The medium was replaced every 2 days. Cells were allowed to differentiate for 8 days when most of the cells had fused into myotubes.

### 2.7. Cell Doubling Time and Colony Efficiency

The cell doubling time (CDT) signifies the period taken for a cell population to undergo a two-fold increase within the logarithmic phase of cellular proliferation. For the assessment of doubling time in cell culture, the cells (*n* = 5) with known concentrations were incubated for 48 h and trypsinized to calculate the cell doubling time. Viable cell counts were determined by the LUNA automated cell counter. The growth rate and CTD were calculated using the following equations.

(1) $$growth\;rate\left(h^{-1}\right)=\frac{ln\left(\frac{viable\;cells\;at\;passaging}{viable\;cells\;at\;seeding}\right)}{culture\;time},$$

(2) $$cell\;doubling\;time\left(h\right)=\frac{ln\left(2\right)}{growth\;rate{(h}^{-1})}.$$

Colony-forming efficiency refers to the quantification of colonies derived from a specific initial cell concentration. OFMC cells, in their 15th passage, were cultivated in 6-well plates with different seeding densities: 50, 100, 200, 500, 1000, and 2000 cells per well with the complete growth medium. Incubation took place at 28 °C, with fresh medium replenished every other day for 14 days. Colonies were manually enumerated with a fluorescence inverted microscope after fixing and staining with anhydrous methanol and Giemsa. This methodology was independently replicated thrice, and the efficiency of colony formation was assessed using the following formula.

(3) $$Colony\;forming\;efficiency=\frac{No.\;of\;colonies}{No.\;of\;cells\;seeded}\times 100.$$

### 2.8. Monitoring the Spontaneous Immortalization of OFMC Cell Line

Primary muscle cells were cultured continuously by subculturing. To establish a cell line without genetic modifications, the OFMC cell line underwent a spontaneous immortalization crisis. This crisis, as previously described by Li et al. (2021) [25], is a large-scale apoptosis/senescence event that results in sporadic clusters of deep-colored (darkened) quiescent cells. These cells then initiate mitosis and begin to grow out of quiescence to reach full confluence. The OFMC cell line underwent such events from passage 16 to passage 20. During this time, there was an increased incidence of apoptotic events and senescent cell morphology. First, a large-scale apoptosis occurred suddenly in OFMC cells on the fourth day of post-subculture. The fibroblast-like cells shrank, rounded up, and clustered; the cellular color darkened, and subsequently, most of the OFMC cells detached or fragmented, suggesting that senescence had occurred in OFMC cells. Apoptosis/senescence was observed through microscopic observations. However, there were also clusters of viable cells that underwent spontaneous immortal transformation. During the spontaneous immortal transformation, by passage 20, the OFMC cell line developed into a continuous cell strain named (olive flounder 20 immortalized muscle cells) OF20IMC.

### 2.9. Whole-Cell Protein Lysate Extraction

Myoblasts were differentiated for 8 days, washed with PBS (Gibco; Thermo Fisher Scientific), and lysed in RIPA lysis buffer (50 mM Tris-HCl, 1 mM EDTA,150 mM sodium chloride, 1% NP-40, and 0.25% sodium deoxycholate; pH 7.4) containing protease inhibitors (Thermofisher; Cat no: 78429; 1 μg/mL aprotinin, 1 μg/mL leupeptin, 1 μg/mL pepstatin, 1 mM sodium orthovanadate, 1 mM sodium fluoride, and 1 mM phenyl-methane-sulfonyl fluoride) on ice. After incubation at 4 °C for 30 min, the extracts were centrifuged at 16,000× *g* at 4 °C for 20 min. The protein concentration of supernatants was determined using a bicinchoninic acid (BCA) protein assay kit (Pierce; Thermo Fisher Scientific, Inc.). The kit includes BCA reagent A, BCA reagent B, and bovine serum albumin (BSA) standard. A series of BSA standards were prepared with known concentrations of 0, 25, 125, 250, 500, 750, 1000, 1500, and 2000 µg/mL. These standards were used to generate a standard curve for protein quantification. The BCA working reagent was prepared by mixing BCA reagent A and BCA reagent B in a 50:1 ratio. Twenty-five microliters (µL) of sample or standard were mixed with 200 µL of BCA working reagent and incubated at 37 °C for 30 min. The absorbance of the solution was measured at 562 nm using a microplate reader. A standard curve was plotted by plotting the absorbance values of the BSA standards against their known concentrations. The protein concentration of the samples was determined by comparing their absorbance values to the standard curve.

### 2.10. Western Blotting

Equal amounts of protein (30 μg) were separated by 12% sodium dodecyl sulfate-polyacrylamide gel electrophoresis and transferred to polyvinylidene fluoride (PVDF) membranes (EMD Millipore). The membranes were blocked by incubation with 3% bovine serum albumin in TBS T (10 mM Tris HCl, 150 mM NaCl, and 0.1% Tween 20) at room temperature for 60 min, and incubated with specific primary antibodies (dilution, 1:1000) overnight at 4 °C with gentle agitation. The membranes were washed thrice (for 15 min each time) in TBS T and incubated for 60 min at room temperature with the appropriate horseradish peroxidase-conjugated secondary antibody diluted 1:10,000 in TBS T. Signals were detected using an Enhanced Chemiluminescence Western Blot Analysis Kit (Thermo Fisher Scientific). Experiments were conducted in triplicate, and densitometry analysis was performed using ImageJ software (NIH, Bethesda, MD, USA). The primary antibodies were MyoD (E-1) (Santa cruz cat number: sc-377186; mouse), myogenin (Santa cruz cat number: sc-12732; mouse), and desmin (Santa cruz cat number: sc-23879; mouse); anti-β-actin (Santa cruz cat number: sc-47778; mouse) antibody was used as the control. The secondary antibody was horse radish peroxidase-conjugated anti-mouse IgG (cat. no. 7076S; Cell Signaling Technology, Danvers, MA, USA).

### 2.11. Fluorescence Staining

To visualize the live and dead cells, calcein AM (Thermofisher; cat. Number: C1430) and Propium Iodide (Thermofisher; cat. Number: P1304MP) staining were used, respectively. Undifferentiated and differentiated cells were fixed in a 4% paraformaldehyde solution for 30 min. They were then washed in 0.1 M phosphate-buffered saline (PBS) and permeabilized for 10 min in a solution of 0.1% Triton X-100, 0.5% bovine serum albumin (BSA), and 20 mM glycine. Next, the cells were blocked for 20 min with 1% BSA in a solution of 0.1% Triton X-100 and 20 mM glycine. To specifically identify myocytes, the cells were stained with actin using a mixture of 1% BSA, 0.1% Triton X-100, 20 mM glycine, and fluorescein isothiocyanate (FITC)-phalloidin (4 μg/mL; Sigma) for 30 min. Nuclei were counterstained with 1 μg/mL of 4′,6-diamidino-2-phenylindole (DAPI; Sigma). Images were acquired at a ×20 magnification using a Zeiss confocal microscope.

### 2.12. Microscopy

Photomicrographs of the bright field images were acquired using the Nikon TS-100 microscope at 10× magnifications. Images were captured and analyzed using NIS-elements software. Fluorescence images (were acquired using a Zeiss confocal microscope. Images were captured and analyzed using Zensoft software.

### 2.13. Statistical Analysis

The results are presented as the mean ± standard deviation of at least three independent experiments. Data were analyzed by two-way analysis of variance followed by Tukey’s multiple comparisons test using GraphPad Prism software version 9.4.1. *p* < 0.05 was considered to indicate a statistically significant difference.

## 3. Results

### 3.1. Primary Cell Isolation

**Figure 1:**
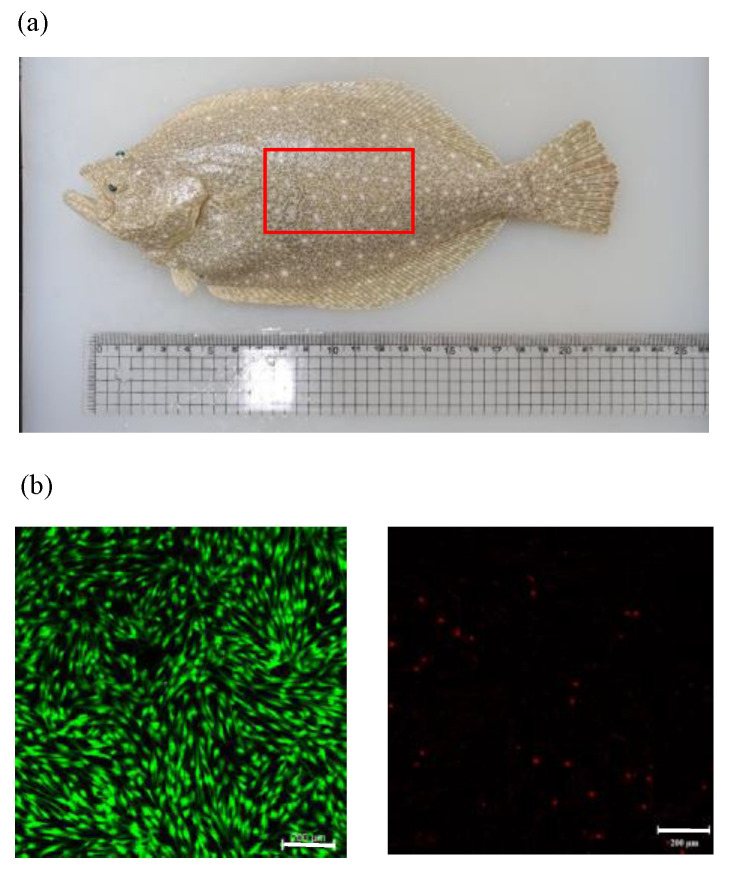
(**a**) *Paralichthys olivaceus* (red color rectangle indicates the place where muscle tissue was collected). (**b**) Live and dead cells of olive flounder muscle cells stained with calcine AM and propidium iodide. Scale bar: 200 µm.

**Figure 2:**
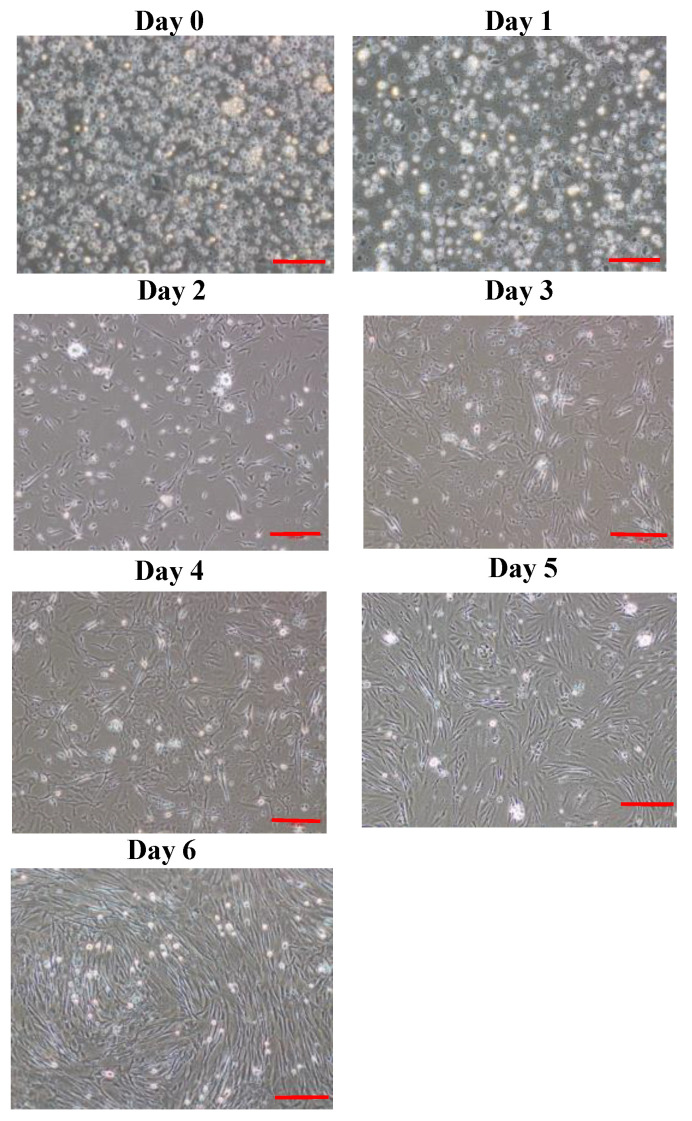
Phase contrast micrographs of olive flounder muscle satellite cells attachment and proliferation from days 0 to 6. Scale bar: 100 µm.

Enzymatic digestion of 5 g of white tissue muscle yielded around 2.88 × 10^6^ cells/mL, of which 2.64 × 10^6^ cells/mL (91.67%) were viable, and 2.40 × 10^5^ cells/mL were non-viable (Figure 1). Cell replication was evidenced by scattered mononuclear cells at day 0 (Figure 2). A shape change in myo-satellite cells was observed from day 1 (Figure 2). The cells reached almost 80% confluence on day 6 (Figure 2). After reaching confluence, the cells were trypsinized or subcultured for up to eight passages.

### 3.2. Explant Primary Cell Isolation

**Figure 3:**
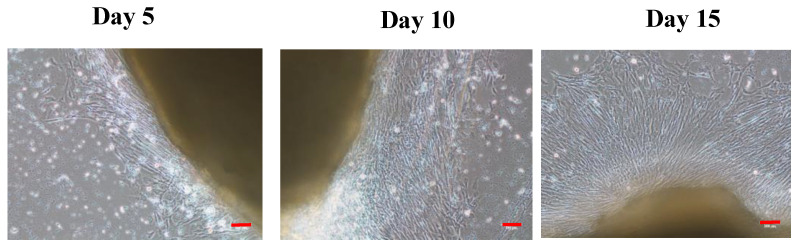
Explant primary cell isolation. Radial movement of cells from explant tissue. Scale bar—100 µm.

Explants of skeletal muscle were affixed after 24 h, and the cells started radiating after 5 days. Cells reached 80% confluence after 15 days (Figure 3).

### 3.3. Optimal Growth Conditions

**Figure 4:**
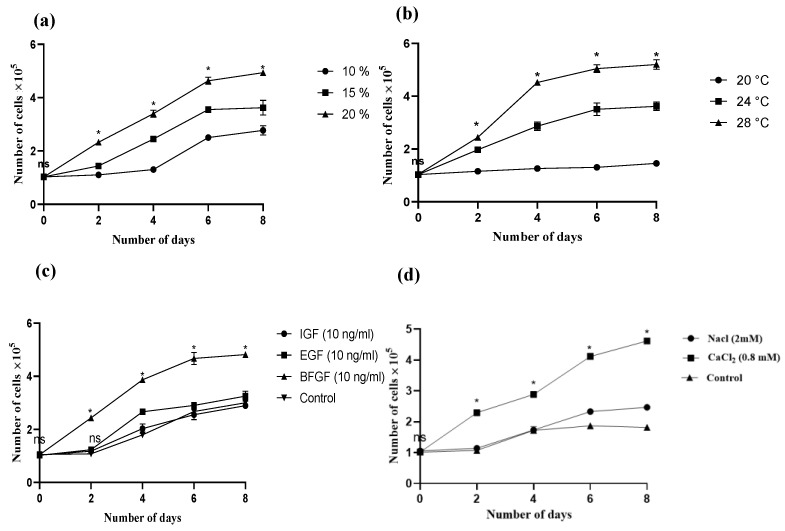
Optimal growth conditions for OFMCs. (**a**) FBS concentration, (**b**) temperature, (**c**) salt concentration, and (**d**) growth factors. ns—non-significant; *—significance (*p* < 0.01).

OFMC growth was lowest in the presence of 10% FBS and highest with 20% FBS, followed by 15% FBS (Figure 4a). Maximum OFMC growth was observed at 28 °C, followed by 24 °C and 20 °C. (Figure 4b). OFMC growth was enhanced by the presence of 0.8 mM CaCl_2_ (Figure 4c). Among the growth factors, BFGF enhanced the growth of OFMCs (Figure 4d).

### 3.4. Cell Doubling Time and Colony Forming Efficiency

The OFMC cell line exhibited an estimated doubling time of 32.21 ± 2.06 h during the 15th passage, and a corresponding colony-forming efficiency of 5 different concentrations were calculated (Table S1) and the average was 15.4 ± 2.6%.

### 3.5. The Spontaneous Immortalization of OFMC Cell Line

**Figure 5:**
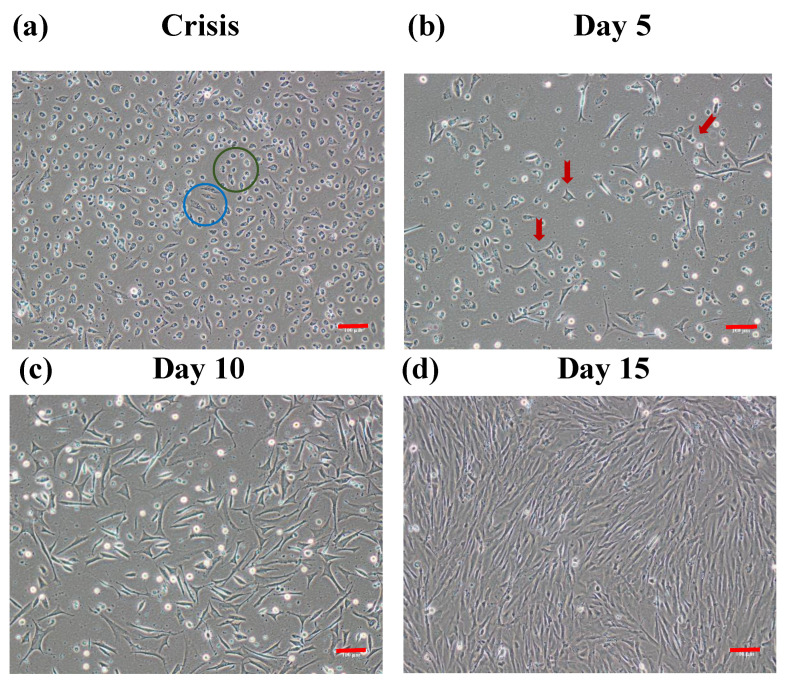
Four representative images of spontaneous immortalization of OFMC cells. (**a**) Large-scale apoptosis and senescence occurred in the OFMC cells. Green circle—sporadic cluster; blue circle—detaching and defragmented cell. (**b**) The remaining cells initiated to overcome the crisis. Red arrow—sporadic cells started proliferating into myocytes. (**c**) Cells began to actively divide again. (**d**) The formation of a confluent cell monolayer Scale bar: 100 µm.

The spontaneous immortalization of the OFMC cell line was characterized by four distinct stages. Initially, a large-scale senescence/apoptosis event occurred after 4 days of post-subculture. The cells began to shrink and form rounded, sporadic clusters (Figure 5a). By the second stage, most of the cells had died, and only a few sporadic cells remained. These cells stopped dividing and remained dormant in the culture flask (Figure 5b). During the third stage, the surviving cells began to adapt to the in vitro conditions and initiated proliferation (Figure 5c). Finally, the actively dividing OFMC cells formed a confluent monolayer (Figure 5d). The newly derived cell line through this spontaneous immortalization event was named (olive flounder 20 immortalized muscle cells) OF20IMC. The OF20IMC cell line exhibited consistent growth and noticeable alterations following the event. These alterations included morphological changes, resulting in shorter and more uniform cells. The alterations were quantified by measuring cell diameters before and after the event: 15.2 µm (±0.43 SD) and 13.5 µm (±0.11 SD), respectively. Notably, the cell doubling time was significantly reduced from 32 h (±8.16 SD) to 28.2 h (±3.82 SD) after the crisis event.

### 3.6. Cell Differentiation

**Figure 6:**
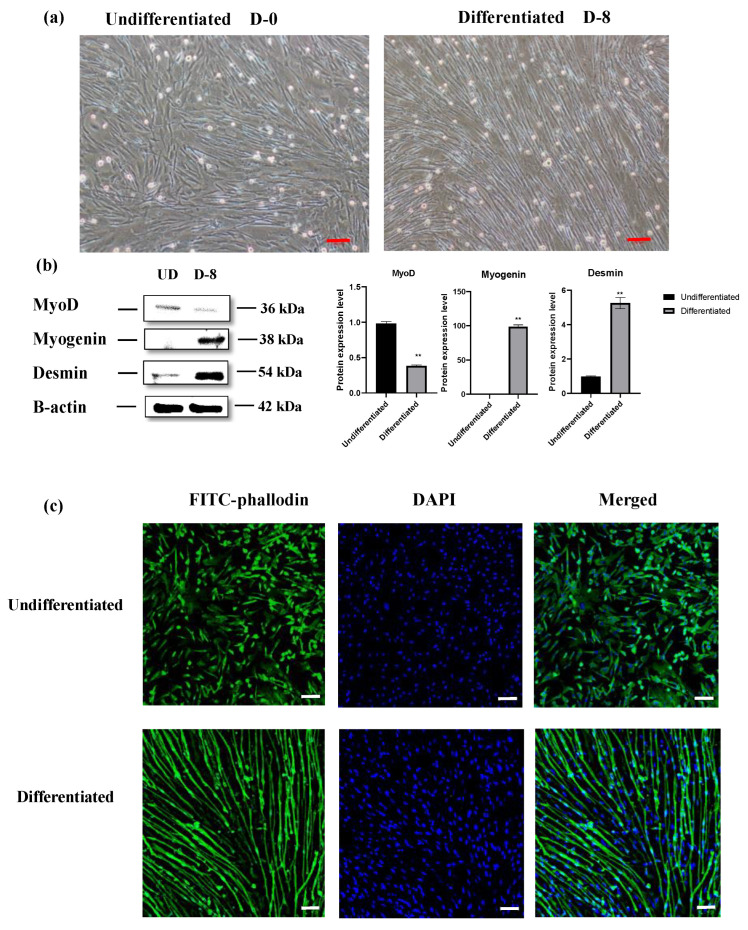
Differentiation of OFMCs into myotubes. (**a**) Representative images of undifferentiated (myoblasts) and differentiated cells (myotubes) incubated with 2% horse serum. Magnification, 10×; Scale bar—100 μm. (**b**) MyoD, myogenin, and desmin protein expression levels in undifferentiated and differentiated cells. Results are means ± standard deviation of three independent experiments. ** *p* < 0.05 vs. corresponding control group (D-0). CON, control; D, day; UD, undifferentiation, D-0. (**c**) FITC-phalloidin and DAPI were used to visualize actin and nucleus staining, and the colocalization of phalloidin and DAPI staining is indicated in the merged images. Magnification 10×; scale bar—100 μm. green- cytoskeleton staining, blue-nucleus staining.

OFMC differentiation was observed through the formation of multinucleated myotubes after 8 days of incubation with 2% horse serum (HS). Enlargement of the cytoskeleton was observed visibly on day 8 compared to day 0 (Figure 6a). Additionally, myogenin and desmin expression, which regulate the terminal differentiation of muscle cells, increased on day 8. MyoD expression was increased in undifferentiated samples and decreased in differentiated samples (Figure 6b and Figure S1). These results indicate the complete differentiation of myoblasts into myotubes after 8 days. Cultivated olive flounder muscle cells were fixed and stained with FITC-phalloidin to visualize F-actin fibers. This revealed significant alterations in actin reorganization between undifferentiated and differentiated states. DAPI staining was used to delineate the positioning of muscle cell nuclei relative to the actin cytoskeleton (Figure 6c).

## 4. Discussion

OFMCs with in vitro culture ability were established and characterized in terms of the isolation method, optimal growth conditions (FBS concentration, temperature, salt conditions, and growth factors), and myogenic protein expression. So far, the cells isolated from olive flounder by enzymatic methods were subcultured up to 30 passages. Skeletal muscle satellite cells were isolated from rainbow trout, Atlantic salmon, Zebrafish, goldfish, humpback grouper, fathead minnow, gilthead sea bream, freshwater catfish, barramundi, bluefin trevally, snakehead, Korean rockfish, turbot, and white sturgeon using enzymatic [26,27,28,29,30,31,32,33,34,35,36] and explant [10,25,37,38,39,40,41,42,43,44,45,46] methods. Previous studies suggested that enzymatic digestion is tedious and time-consuming, which could affect cell viability and lead to different muscle portions and possible contaminations [47,48,49]. Some studies have found that explant primary cell culture can eliminate these disadvantages and protect and improve cell viability [10,50]. However, our results suggest that fast and stable growth was observed from enzymatic cell isolations compared to the explant method. In the explant method, the cells began to radiate after 5 days of incubation, but the growth was very slow, taking 15 days to reach confluence (Figure 3). In contrast, the cells reached confluence in only 6 days using the enzymatic digestion method (Figure 2). These results suggest that enzymatic digestion is a promising method for muscle cell isolation, as it is faster, more stable, and produces a higher cell yield than the explant method.

FBS is an essential medium component for cell survival and growth. In this study, 20% of FBS maximized cell growth and attachment. Optimal growth was observed with 15% FBS, which could enable long-term maintenance of cells at a low cost. These results agree with prior reports of the optimal FBS concentration for fish cell culture [31,40,41,51]. Despite being the most commonly used medium supplement in cell culture, FBS has limitations for use in cultivated food production due to several factors, including high cost, potential contamination, limited supply, batch-to-batch variation, inability to grow specific cells, animal welfare concerns, environmental impact, and high protein content [20]. These limitations can be countered by optimizing a cost-effective serum-free medium that applies sustainable protein sources. This will increase the yield and similarity to the phenotypic characters of target seafood cells. In view of this, efforts will be made to analyze the effect of different compounds on the cell and decide on their concentration. Fish cells can grow at temperatures of 18–32 °C depending on the warm or cold water-adapted fish type [30,52,53,54,55]. Maximum OFMC proliferation occurred at 28 °C in this study, consistent with findings in marine [56] and freshwater [41,57] cell lines. 

The osmolality of L-15 medium is 300–340 mOsmkg^−1^, and after standard preparations of L-15/exposure (L-15/ex), which consisted of L-15 salts, galactose, amino acids, vitamins, and pyruvate dissolved in commercial tissue culture water (Gibco/BRL) the mean value was in the range of 326 mOsmkg^−1^ T 9 (*n* = 8) [58]. The osmolality of marine fish blood is generally from 250 to 400 mOsm/kg, and marine fish cell lines survive well within this range. Osmolality can be adjusted by adding or removing salts [59]. Previous studies have suggested that enriching salt concentrations enhances the growth of fish cell lines. For example, a study on the caudal fin cell line of the ornamental fish *Amphiprion ocellaris* found that better cell growth was observed with the addition of 1% of 0.2 M (2 mM) NaCl [59]. However, another study on the zebrafish *Danio rerio* muscle cell line found that the addition of 0.8 mM CaCl_2_ was more effective [26]. However, L-15 media already contains 137.9 mM NaCl and 1.26 mM CaCl_2_. To understand the effect of slightly increasing the concentration of these salts, we conducted an experiment. Our results showed that slightly increasing the CaCl_2_ concentration from 1.26 mM to 2.01 mM (by adding 0.8 mM CaCl_2_) enhanced fish cell growth by regulating osmolality. This was more effective than enriching the media with NaCl (from 137.9 to 139.9 mM) or using media without additional salt. Additionally, the addition of bFGF (10 ng/mL) enhanced cell growth over that induced by IGF (10 ng/mL) and EGF (10 ng/mL). Further, the plating efficiency was found to be lower, and the maximum plating efficiency was observed with a concentration of 2000 cells per ml (19.8%). The average plating efficiency of OFMC (15.4 ± 2.6%) was consistent with those calculated for muscle cells of *labia rohita* [60] and *Carassius auratus* [43]. Previous studies also suggested that supplementation with bFGF has a positive effect on fish muscle cell culture. These studies also suggest that the reduction or absence of bFGF will have an effect on the proliferation of fish cell growth. [8,41,59,60]. OFMC differentiations were induced by inducing serum starvation by replacing FBS with 2% horse serum (HS), as used in the previous literature [10]. A previous study suggested 8% HS for the differentiation of croaker muscle cells. At the same time, the induction of OFMC differentiation was observed with just 2% HS. The low frequency of myogenic differentiation and cell death during serum starvation can be attributed to the need for high horse serum (HS) concentrations for differentiation in Croaker (PSC) muscle cells. On the other hand, OFMC cells can achieve high frequencies of myogenic differentiation and low cell death during serum starvation, even with 2% HS [61].

Our cell line successfully passed through an episode of spontaneous immortalization crisis and has continued to proliferate continuously since then. The use of spontaneous immortalization as a technique for establishing a cell line is preferred over genetic modification methodologies, given the heightened regulatory scrutiny that genetically modified organisms (GMOs) face, particularly in the European context [24]. To date, the availability of continuous fish muscle cell lines established through immortalization crisis without genetic modifications is limited [24,25]. This highlights the potential significance of our current cell line for applications in cellular aquaculture and other cell line-based applications. Cell-based fish production relies on four key factors: the appropriate muscle cell type, optimized cell culture conditions, large-scale production in a bioreactor, and the growth and differentiation of cells in a scaffold. However, our understanding of the molecular mechanisms that control the proliferation and differentiation of mononuclear myoblasts to multinucleated myotubes is limited, hampering cell-based fish meat production. [18]. Myogenic regulation factors (MRFs) such as PAX7, MyoD, Myf5, and Myogenin are crucial genes for controlling cell activation, proliferation, and differentiation for muscle plasticity [62]. Myf5 and MyoD are typically expressed in proliferating myoblasts depending on the distinct cell cycle regulation. Myogenin and MyoD level is upregulated shortly after the commencement of differentiation. Soon after, Myf5 and MyoD levels gradually decrease, while myogenin levels are increased during the differentiation process [63]. Desmin is a muscle-specific member of the intermediate filament protein family and was the first myogenic marker discovered in muscle and heart. It is expressed in all known muscle proteins, including members of the MyoD family [64]. In this study, we assessed levels of MRF such as MyoD, myogenin, and desmin. The findings were in line with the literature, where MyoD was highly expressed in undifferentiated cells and decreased over the differentiation process. At the same time, myogenin and desmin were observed in higher amounts in differentiated cells (Figure 6b and Figure S1).

Olive flounder (*Paralichthys olivaceus*) is a commercially important fish species in aquaculture. Establishing a cell line allows for the in-depth study of its physiological processes, disease susceptibility, and immune responses at the cellular level. Cultivating cells in vitro reduces the need for large numbers of live fish for research purposes. This conservation-oriented approach helps protect wild populations and ecosystems. The established cell line should possess the ability to proliferate and maintain its characteristics over prolonged periods in culture, ensuring its usefulness as a long-term research tool. A stable cell line allows for continuous cultivation, providing a consistent source of cells for experimentation without the need for continuous collection from live animals.

## 5. Conclusions

The OFMCs cell line was successfully developed and characterized by optimal growth conditions (FBS concentration, temperature, salt conditions, and growth factors). Muscle satellite cells were confirmed with the different myogenic protein expressions (MyoD, myogenin, and desmin). We validated the optimal growth conditions and myogenic regulation factors. In conclusion, the isolation of an olive flounder cell line is a crucial step toward advancing research and development efforts in aquaculture, with far-reaching implications for fish health, disease management, and sustainable aquaculture practices. This invaluable tool contributes to the conservation of wild populations and fosters innovation in biotechnological applications for this economically important species. Moreover, cellular aquaculture mainly depends on developing and characterizing suitable cell lines that are economically important and preferred aquaculture species. Future work will focus on improving culture techniques to grow cells and tissues in a synthetic environment (scaffolds, cell sheets, and 3d spheroid), promoting their survival, proliferation, and growth, creating serum-free media formulations using sustainable protein sources, studying myogenesis, and differentiating the developed continuous cell line.

## Data Availability

The datasets used or analyzed during the current study are available from the corresponding author upon reasonable request.

## Supplementary Materials

The following supporting information can be downloaded at: https://www.mdpi.com/article/10.3390/cells12182325/s1, Figure S1: Unprocessed western blot images of different protein expression. UD-undifferentiated DI-differentiated; Table S1: Colony forming efficiency of OFMC cell line at different concentrations.
