# Supplementary material for: Establishment and Characterization of Continuous Satellite Muscle Cells from Olive Flounder (Paralichthys olivaceus): Isolation, Culture Conditions, and Myogenic Protein Expression
Source: Cells. 2023 Sep 21;12(18):2325. doi: 10.3390/cells12182325 (PMC10527956; doi:10.3390/cells12182325)
Supplement: Supplementary file 1 [file cells-12-02325-s001.zip › cells-2580761-supplementary.pdf]

Supplimentary files

Supplementary Table S1. Colony forming efficiency of OFMC cell line at different concentrations.

Concentration	Colonies	CFE
50	7	14 %
100	12	12 %
200	31	15.5 %
500	68	13.6 %
1000	176	17.6 %
2000	396	19.8 %

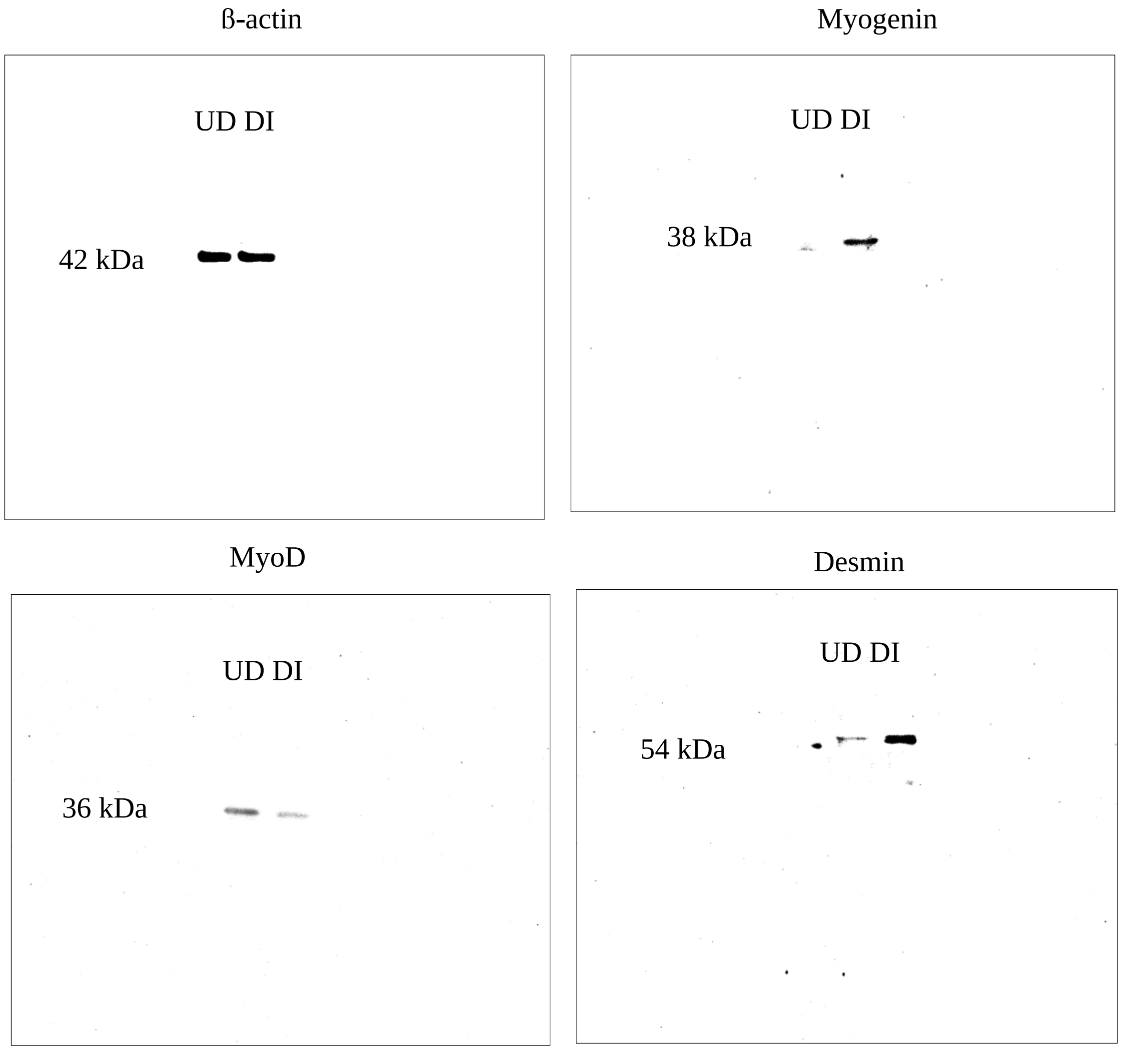

Supplementary Figure S1. Unprocessed western blot images of different protein expression. UD-undifferentiated DI-differentiated.
